# Cancer detection rate of prebiopsy MRI with subsequent systematic and targeted biopsy are superior to non-targeting systematic biopsy without MRI in biopsy naïve patients: a retrospective cohort study

**DOI:** 10.1186/s12894-018-0361-4

**Published:** 2018-05-30

**Authors:** Satoshi Washino, Shigeru Kobayashi, Tomohisa Okochi, Tomohiro Kameda, Tsuzumi Konoshi, Tomoaki Miyagawa, Tatsuya Takayama, Tatsuo Morita

**Affiliations:** 1 Department of Urology, Jichi Medical University, 3311-1 Yakushiji, Shimotsuke, Tochigi, 329-0498 Japan; 2 Department of Radiology, Jichi Medical University, 3311-1 Yakushiji, Shimotsuke, Tochigi, 329-0498 Japan; 3 Department of Urology, Jichi Medical University Saitama Medical Center, 1-847, Amanuma-cho, Omiya-ku, Saitama, 330-8503 Japan; 4 Department of Radiology, Jichi Medical University Saitama Medical Center, 1-847, Amanuma-cho, Omiya-ku, Saitama, 330-8503 Japan

**Keywords:** Magnetic resonance imaging, Biopsy, Target, Random, Prostate cancer

## Abstract

**Background:**

To determine whether prebiopsy multiparametric magnetic resonance imaging (mpMRI) with subsequent systematic plus targeted biopsies for suspicious lesions improve prostate cancer detection compared with standard non-targeting systematic biopsies without mpMRI in biopsy-naïve patients.

**Methods:**

Patients who underwent their first prostate biopsy due to suspicion of prostate cancer were analyzed retrospectively to compare the biopsy outcomes between patients who received prebiopsy mpMRI (215 patients) and those who did not (281 patients). mpMRI was performed to determine pre-biopsy likelihood of the presence of prostate cancer using a three-point scale (1 = low level of suspicion, 2 = equivocal, and 3 = high level of suspicion). Systematic biopsies were performed in both groups. Targeted biopsies were added for a high level of suspicious lesions on mpMRI. All biopsies were performed by transperineal biopsy technique. After biopsy, Prostate Imaging Reporting and Data System ver. 2 (PIRADS-2) scoring was performed to describe the mpMRI findings and predictive value of PIRADS-2 was evaluated.

**Results:**

The detection rate of total and clinically significant prostate cancer was significantly higher in patients who received prebiopsy mpMRI than in those who did not (55.3 and 46.0% vs. 42.0 and 35.2%, respectively; *p* = 0.004 and *p* = 0.016). The clinically insignificant prostate cancer detection rate was similar between the two groups (9.3% vs. 6.8%; *p* = 0.32). Of 86 patients who underwent systematic plus targeted biopsy in the MRI cohort and were diagnosed with prostate cancer, seven patients were detected by addition of targeted biopsy whereas 29 patients were missed by targeted biopsy but detected by systematic biopsy. There was a correlation between the PIRADS-2 and prostate cancer detection rate, and a receiver-operator curve analysis yielded an area under the curve of 0.801 (*p* <  0.0001).

**Conclusions:**

Prebiopsy mpMRI with subsequent systematic plus targeted biopsies for suspicious lesions can yield a higher cancer detection rate than non-targeting systematic biopsies. PIRADS-2 scoring is useful for predicting the biopsy outcome.

## Background

Prostate cancer (PCa) is the most common male malignancy and the second most common cause of male cancer-related death [1]. It is usually diagnosed based on systematic transrectal ultrasound (TRUS)-guided random biopsies of the prostate gland. However, a significant number of transrectal biopsies are negative for cancer, yielding inaccurate results [2–4]. The cancer detection rate with a standard TRUS-guided prostate biopsy is only 20~ 40% [5]. Furthermore, TRUS-guided transrectal biopsies have a limited ability to sample the anterior prostate [6].

The ideal prostate biopsy goal would be to identify clinically significant PCa and minimize the detection of indolent disease. The growing availability of prostate multiparametric magnetic resonance imaging (mpMRI), novel functional imaging modalities, and increased standardization have created an opportunity for the detection, localization, and staging of PCa [7]. Screening patients using mpMRI may avoid the morbidity associated with a biopsy if no lesions are seen [8]. In addition, targeted biopsies should identify more clinically significant PCa than non-targeted TRUS-guided biopsies [9]. High PCa detection rates have been reported using magnetic resonance imaging (MRI)-targeted biopsies, both in patients with prior negative TRUS biopsies and in biopsy-naïve patients [9–13]. However, it is not clear whether prebiopsy MRI with a subsequent targeted biopsy is superior to the traditional systematic non-targeted TRUS biopsy in biopsy-naïve patients [14–16]. Therefore, this study examined whether prebiopsy mpMRI with the subsequent addition of targeted biopsies for suspicious lesions could improve the PCa detection rate in biopsy-naïve patients. These results were compared with those of a standard cohort of patients who underwent systematic non-targeted TRUS biopsies without MRI.

## Methods

### Patients

This retrospective observational study was approved by the local Institutional Review Board. The eligibility criteria were as follows: patients with a prostate-specific antigen (PSA) level < 15 ng/mL who underwent their first prostate biopsy for suspected PCa at Jichi Medical University or Jichi Medical University Saitama Medical Center between January 2010 and April 2014. Patients who underwent mpMRI before their prostate biopsy were assigned to the MRI cohort, whereas those who did not undergo MRI were assigned to the non-MRI cohort. Physicians decided who was to undergo MRI as their beliefs and patients’ preferences after discussion with patients. A total of 557 patients were eligible: 383 at Jichi Medical University and 174 at Jichi Medical University Saitama Medical Center. Sixty-one patients were excluded based on the following criteria: interval from mpMRI to biopsy > 6 months (25 patients), < 12 biopsy cores (17 patients), MRI performed in another hospital (11 patients), the use of 5α-reductase inhibitors or anti-androgen therapy at the time of biopsy (6 patients), previous prostate surgical intervention (1 patient), and a blurred MRI scan (1 patient). Data from 496 patients were analyzed, and 215 and 281 patients were assigned to the MRI and non-MRI cohorts, respectively.

### Imaging

All patients in the MRI cohort underwent mpMRI, which was performed using a 1.5-Tesla (Excelart Vantage, Toshiba Medical Systems, Otawara, Japan; MAGNETOM Symphony Advanced, Siemens, Munich, Germany; MAGNEOM Avanto, Siemens; or Achieva, Philips, Amsetrdam, Netherlands) or 3-Tesla (Vantage Titan 3 T, Toshiba Medical Systems; or MAGNETOM Skyra, Siemens) machine with a 16-channel phased-array body coil. The protocol included T2-weighted imaging, diffusion-weighted imaging, and dynamic contrast-enhanced imaging. Radiologists evaluated the mpMRI results and determined the locations of suspicious lesions. The likelihood of the presence of PCa was determined using a three-point scale (1 = low level of suspicion, 2 = equivocal, and 3 = high level of suspicion) because the standardized Prostate Imaging Reporting and Data System (PIRADS) criteria were not used to evaluate the images when the biopsies were performed. However, one very experienced genitourinary radiologist (T.O. or S.K.) at each institute blinded to the biopsy and the first MRI evaluation before biopsy reviewed and scored each suspicious lesion in the mpMRI image from 1 to 5 points according to the PIRADS criteria (ver. 2.0; PIRADS-2) [17, 18]. PI-RADS is both quantitative and qualitative, but only qualitative scoring was used in this study. The highest overall PIRADS-2 score of each mpMRI scan was used.

### Biopsy protocol

**Fig. 1:**
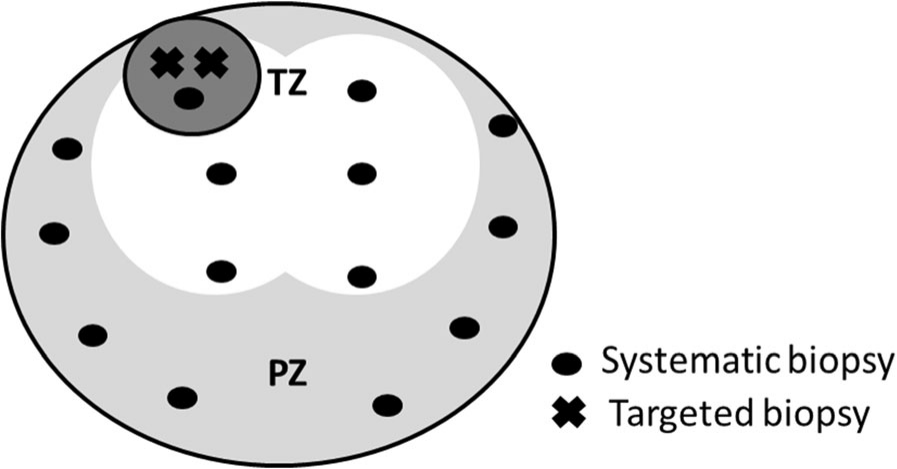
Biopsy strategy. In the MRI cohort, 12 to 14 cores were biopsied. In patients who had suspicious lesions on mpMRI, each suspicious lesion could be targeted as one of systematic biopsy at the nearest point and further typically two targeted biopsies were added for each lesion. White, light gray, and dark gray areas indicate transitional zone, peripheral zone of axial view, and index lesion on mpMRI, respectively. Black dot indicates systematic biopsy cores and x indicates targeted biopsy cores. TZ; transitional zone, PZ: peripheral zone

All biopsies were performed using a transperineal approach with an 18-gauge needle biopsy gun under general or spinal anesthesia. In the non-MRI cohort, 12 to 14 cores were biopsied during non-targeted systematic TRUS-guided transperineal biopsies. In the MRI cohort, 12 to 14 cores were also biopsied during systematic TRUS-guided transperineal biopsies. However, in patients who had suspicious lesions on mpMRI, each lesion was targeted in one of the systematic biopsies and, typically, two cores of targeted biopsies were added for each lesion (Fig. 1). The targeted biopsies were performed using the cognitive registration technique described previously [19], with a minor modification: we used an ultrasound-guided freehand biopsy instead of a transperineal template.

### Clinically significant cancer

Clinically significant PCa was defined as a Gleason score (GS) ≥ 3 + 4 or a maximum cancer core length ≥ 4 mm; all other lesions were deemed as clinically insignificant PCa. This threshold has been validated to predict lesions with tumor volumes ≥2 mL [19].

### Study endpoints

Patients’ characteristics and cancer detection rate were compared between the MRI and non-MRI cohort. The endpoint of this study was the detection rate of all PCa and clinically significant PCa.

### Statistical analysis

The data were analyzed using the Mann–Whitney *U* test or Fisher’s exact test. Univariate and multivariate analyses were performed using logistic regression analysis to determine significant predictors of PCa. A *p*-value ≤ 0.05 was considered significant. The statistical analyses were performed using GraphPad Prism (ver. 5.0; GraphPad, La Jolla, CA, USA) and SPSS for Windows software (ver. 19.0; SPSS Inc., Chicago, IL, USA).

## Results

### Patient characteristics, MRI images, and biopsy strategy

**Fig. 2:**
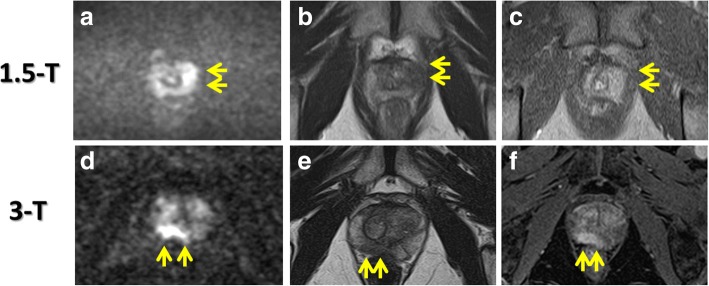
MRI images. 1.5-Tesla MRI images obtained from a patient with a PSA level of 3.86 ng/mL (A − C). Diffusion weighted image (DWI) with a b-value 1500 s/mm^2^ (A) showed a high intensity area in the left peripheral zone (Arrow). T2 weighted image (T2WI: B) and dynamic contrast enhancement image (DCEI: C) showed a low intensity area and an enhancement, respectively, in the same lesion, which was considered to be a high level of suspicious. 3-Tesla MRI images obtained from a patient with a PSA level of 3.57 ng/mL (D − F). DWI with a b-value 2000 s/mm^2^ (D) showed a high intensity area in the right peripheral zone (Arrow). T2WI (E) and dynamic DCEI (F) showed a low intensity area and an enhancement, respectively, in the same lesion, which was considered to be a high level of suspicious

There were no significant differences in patient characteristics between the two cohorts, except for prostate volume, which was significantly smaller in the MRI cohort than in the non-MRI cohort (median = 27.7 vs. 32.0 cm^3^, *p* = 0.0002; Table 1). Examples of 1.5- and 3-Tesla MRI images are shown in Fig. 2. The 3-Tesla MRI seems to show the suspicious lesion clearly, compared to 1.5-Tesla MRI. Systematic biopsies were performed in all patients and the median number of cores collected was 12 [interquartile range (IQR) = 12–14] in both cohorts. Targeted biopsies for suspicious lesions on mpMRI were performed in 129 patients (60.0%) in the MRI cohort; 345 cores were targeted for 145 suspicious lesions; the median targeted core number per prostate was two (IQR = 2–4).

**Table 1:** Patient characteristics and biopsy outcomes

	MRI (+) *n* = 215		MRI (−) *n* = 281		*p* Value
	Median	IQR	Median	IQR	
Age	68	(62–72)	68	(63–72)	0.21
PSA (ng/mL)	6.4	(5.2–8.8)	6.7	(5.5–9.4)	0.25
Prostate volume (cm^3^)	27.7	(21.0–36.0)	32.0	(23.0–45.8)	0.0002
PSA density (ng/mL/cm^3^)	0.22	(0.16–0.34)	0.23	(0.16–0.34)	0.79
DRE positive, n (%)	44	(20.5)	41	(14.6)	0.09
TRUS positive, n (%)	32	(14.9)	29	(10.3)	0.13
MRI type, n (%)					
1.5 Tesla	161	(74.9%)	(−)		
3 Tesla	54	(25.1%)	(−)		
Prostate cancer, n (%)	119	(55.3%)	118	(42.0%)	0.004
Gleason sum, n (%)					
3 + 3	34	(15.8%)	42	(14.9%)	0.80
3 + 4	43	(20.0%)	40	(14.5%)	0.08
4 + 3	16	(7.4%)	11	(3.9%)	0.11
8 or more	26	(12.1%)	25	(8.9%)	0.30
Clinical significance, n (%)					
Insignificant cancer	20	(9.3%)	19	(6.8%)	0.32
Significant cancer	99	(46.0%)	99	(35.2%)	0.016
Cancer positive cores	3	(1.25–5)	2	(1–4)	0.28

### Cancer detection rate

The cancer detection rate was significantly higher in the MRI cohort than in the non-MRI cohort (55.3% vs. 42.0%, *p* = 0.004; Table 1). When cancer grade was compared between the two cohorts, there was no significant difference in the detection rate of low-grade cancer (GS 3 + 3; 15.8% in the MRI cohort vs. 14.9% in the non-MRI cohort, *p* = 0.80). However, detection of intermediate- or high-grade cancer (GS ≥ 3 + 4) was significantly higher in the MRI cohort compared with the non-MRI cohort (39.5% vs. 27.0%, respectively, *p* = 0.004). The clinically significant PCa detection rate was also higher in the MRI cohort than in the non-MRI cohort (46.0% vs. 35.2%, respectively, *p* = 0.016).

### Systematic and targeted cores in each cohort

Table 2 summarizes the outcomes of the systematic and targeted cores in each cohort. The rate of cancer-positive cores and cores including a Gleason pattern of 4 or more was significantly higher in targeted biopsies than in systematic biopsies in the MRI and non-MRI cohorts, respectively (31.3 and 23.1% vs. 12.3 and 8.4%, and 10.0 and 5.9%, all *p* <  0.0001) (Table 2). The cancer-positive core rate for systematic biopsies in the MRI cohort was also significantly higher than that in the non-MRI cohort (12.3% vs. 10.0%, *p* = 0.004). The percentage of core length involved by cancer in targeted cores was also significantly higher than that in systematic biopsies, in both the MRI and non-MRI cohorts [median (IQR) = 50% (20–70) vs. 30% (10–60) and 20% (10–50), *p* = 0.0011 and *p* <  0.0001].

**Table 2:** Systematic and targeted cores in each cohort

	MRI (+)				MRI (−)		*p* Value		
	Systematic cores *n* = 2696 (A)		Targeted cores *n* = 345 (B)		Systematic cores *n* = 3542 (C)		A vs B	A vs C	B vs C
Cores of cancer, n (%)	333	(12.3)	108	(31.3)	355	(10.0)	< 0.0001	0.004	< 0.0001
Cores including GP 4 or more, n (%)	229	(8.4)	80	(23.1)	210	(5.9)	< 0.0001	0.0001	< 0.0001
Median percentage of core length involved by cancer (IQR)	30	(10–60)	50	(20–70)	20	(10–50)	0.0011	0.10	< 0.0001

### Addition of targeted biopsies to systematic biopsies

**Fig. 3:**
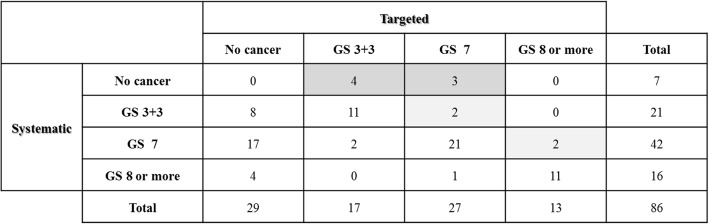
Cross-tabulation of histology (Gleason score) of targeted and systematic biopsy among patients who received both biopsies and had prostate cancer. Seven patients were diagnosed with prostate cancer by addition of targeted biopsies (dark gray box). Additionally, four patients were upgraded to intermediate or high grade by addition of targeted biopsies (light gray box). GS, Gleason score

Of the 129 patients who underwent systematic and targeted biopsies in the MRI cohort, 86 had PCa. We performed a subgroup analysis on these patients to assess the performance of the targeted biopsies (Fig. 3). Seven and four patients were diagnosed with PCa and upgraded to an intermediate and high grade, respectively, by the addition of targeted biopsies. The index lesions in seven patients missed by systematic biopsies but detected by targeted biopsies were in the anterior transitional zone (*n* = 4), anterior stroma (*n* = 1), anterior peripheral zone (*n* = 1), and posterolateral peripheral zone (*n* = 1). Twenty-nine patients were missed by targeted biopsies but detected by systematic biopsies.

### Factors predicting prostate cancer detection

Univariate and multivariate analyses were performed to identify predictors of PCa detection (Table 3). Multivariate logistic regression analysis revealed that PSA level, prostate volume, performing prebiopsy mpMRI, and digital rectal examination findings were independent predictors of PCa detection.

**Table 3:** Univariate and multivariate analysis for prostate cancer detection in all patients

			Univariate analysis			Multivariate analysis		
		N	HR	95% CI	*p*-value	HR	95% CI	*p*-value
Age	≤ 65	189	–	–	–	(−)	(−)	0.101
	66–70	147	1.166	0.755–1.801	0.488			
	> 70	160	1.836	1.119–2.812	0.005			
PSA	≤ 5.0	89	–	–	–	–	–	–
	5.01–7.50	219	1.194	0.721–1.978	0.491	1.346	0.767–2.363	0.300
	7.51–10	97	1.949	1.085–3.499	0.026	2.620	1.342–5.115	0.005
	> 10.0	91	2.712	1.484–4.955	0.001	3.947	1.995–7.806	< 0.001
Prostate volume	≤ 25.0	176	–	–	–	–	–	–
	25.1–35.0	147	0.361	0.228–0.571	< 0.001	0.336	0.205–0.551	< 0.001
	> 35.0	173	0.135	0.084–0.217	< 0.001	0.112	0.073–0.204	< 0.001
MRI	No	281	–	–	–	–	–	–
	Yes	215	1.712	1.197–2.450	0.003	1.749	1.160–2.636	0.008
DRE	Negative	411	–	–	–	–	–	–
	Positive	85	4.193	2.467–7.126	< 0.001	3.068	1.704–5.526	< 0.001
TRUS	Negative	435	-	-	-	(−)	(−)	0.154
	Positive	61	2.500	1.418–4.407	0.002			

### Cancer detection rate according to PIRADS-2 score

The detection rate of all PCa and clinically significant PCa, stratified according to PIRADS-2 scores of 1, 2, 3, 4, and 5 in the MRI cohort, was 31.8 and 18.2%, 9.1 and 9.1%, 14.5 and 10.9%, 78.3 and 66.0%, and 83.3 and 80.0%, respectively (Table 4). The detection rate of clinically significant cancer was significantly higher in patients with a PIRADS-2 score of 4 or 5 compared with those with a PIRADS-2 score of 1–3 (69.3% vs. 12.5%; *p* <  0.0001). By contrast, there was no significant difference in the detection rate of clinically insignificant PCa between groups (10.2% vs. 5.7%; *p* = 0.32). When a PIRADS-2 score of 4 or 5 was considered positive, the sensitivity, specificity, positive predictive value (PPV), and negative predictive value (NPV) for PCa detection were 0.87 [95% confidence intervals (CI), 0.79–0.92], 0.75 (95% CI, 0.65–0.83), 0.81 (95% CI, 0.73–0.88), and 0.82 (95% CI, 0.72–0.89), respectively. Receiver operating characteristic (ROC) curve analysis for predicting PCa detection using the PIRADS-2 score revealed an area under the curve (AUC) of 0.801 (95% CI, 0.738–0.864), which was superior to AUC of 0.738 (95% CI, 0.670–0.806) in the three-point scale at the first radiological evaluation before biopsy.

**Table 4:** Cancer detection rate according to PI-RADS v2 score

	PI-RADS v2 score				
	1 *n* = 22	2 *n* = 11	3 *n* = 55	4 *n* = 97	5 *n* = 30
Insignificant PCa, n (%)	3 (13.6%)	0 (0%)	2 (3.6%)	12 (12.4%)	1 (3.3%)
Significant PCa, n (%)	4 (18.2%)	1 (9.1%)	6 (10.9%)	64 (66.0%)	24 (80.0%)
Total PCa, n (%)	7 (31.8%)	1 (9.1%)	8 (14.5%)	76 (78.3%)	25 (83.3%)

## Discussion

In this study, the detection rate of clinically significant PCa was significantly higher in the MRI cohort than the non-MRI cohort, whereas the clinically insignificant PCa detection rate was similar in both groups (Table 1). Performing MRI was an independent predictor of PCa detection (Table 3). These results suggest that prebiopsy MRI has the potential to improve biopsy outcomes in biopsy-naïve patients. We believe that a prebiopsy MRI has at least two advantages: patients can be selected more efficiently and a targeted biopsy of the index lesion can be added.

Traditionally, the decision regarding whether a prostate biopsy should be performed has been based mainly on the PSA, digital rectal examination findings, and age, which leads to inaccurate results. MRI can provide more precise information about the likelihood of the presence of PCa and prostate volume before biopsy. The prostate volume is negatively associated with the cancer detection rate, as shown in this (Table 3) and previous studies [20] and prostate volume was significantly smaller in the MRI cohort than the non-MRI cohort in this study (Table 1). These suggest that prostate biopsies might not be recommended in patients with a large prostate volume and/or normal MRI. Patient selection may partially explain the higher cancer detection rate in the MRI cohort. The combination of MRI findings and other biomarkers may better determine which patients should undergo prostate biopsy. Recently, we reported that the combination of PIRADS-2 score and PSA density was useful for decision-making before a prostate biopsy [21]. In that study, no patients with a PIRADS-2 score of ≤ 3 and PSA density of < 0.15 ng/mL/cm^3^ were diagnosed with clinically significant PCa. In the present study, 27 patients had a PIRADS-2 score of ≤ 3 and PSA density of < 0.15 ng/mL/cm^3^. Of these, no patients were diagnosed with clinically significant PCa (data not shown).

In this study, targeted biopsies based on MRI detected PCa more efficiently than systematic biopsies (Table 2). Furthermore, some patients in the MRI cohort were diagnosed with PCa or upgraded by the addition of targeted biopsies (Fig. 3). In particular, targeted biopsy was beneficial for the diagnosis of anterior cancer because most of the tumors detected by targeted biopsies, but missed by systematic biopsies, were located in the anterior zone. Collectively, the addition of a targeted biopsy might improve the biopsy outcome. However, the effectiveness of targeted biopsy in this study may have been underestimated because the urologist was not blinded to the suspicious lesions on MRI, so that these could be targeted in systematic biopsies. This suggestion is supported by the fact that the cancer-positive core rate in systematic biopsies in the MRI cohort was significantly higher than that in the non-MRI cohort (Table 2). However, performing the study with the urologists blinded to the MRI during the systematic biopsies would be preferred to evaluate the effectiveness of systematic and targeted biopsies accurately. The cancer detection rate of 55.3% in the MRI cohort in this study was somewhat lower than that reported in previous series that performed targeted biopsies with or without systematic biopsies (56–64%) [10, 13, 22, 23]. Furthermore, one third of the tumors were missed by targeted biopsies, but detected by systematic biopsies (Fig. 3). Three techniques have been reported for targeted biopsies: MRI-guided in-bore biopsy; MRI-TRUS fusion-guided biopsy; and cognitive registration. Although few direct comparisons have been performed, MRI-guided in-bore and MRI-TRUS fusion-guided biopsies likely yield a higher detection rate of clinically significant PCa compared with cognitive registration [13, 14, 22–24]. The cognitive registration technique was used in this study, which may have caused the somewhat low PCa detection rate. However, MRI-guided in-bore and MRI-TRUS fusion-guided biopsies have limited availability and are complex and costly to introduce and/or perform; therefore, they are not feasible for routine use so far. These suggest that targeted biopsies using cognitive registration are more practical and may improve the performance of prostate biopsy.

This study found a correlation between the PIRADS-2 score and PCa detection rate, especially in clinically significant PCa. ROC analysis revealed an AUC of 0.801 which was superior to that of 0.738 in the three-point scale; the sensitivity, specificity, PPV, and NPV were 0.87, 0.75, 0.81, and 0.82, respectively. A meta-analysis assessing the performance of mpMRI for detecting PCa found a specificity of 0.88 (95% CI, 0.82–0.92), sensitivity of 0.74 (95% CI, 0.66–0.81), and NPV of 0.64–0.94, which is consistent with our results. Recently, PIRADS was annotated, revised, and published as a second version, PIRADS-2, to define standards of high-quality clinical service for mpMRI, including image creation and reporting [17, 18]. Kuru et al. performed ROC analysis for PCa detection using the PIRADS score and found an excellent specificity of 0.90–0.98 [25]. Grey et al. [26] reported that a ROC analysis of clinically significant PCa yielded an AUC of 0.88–0.89 and sensitivity, specificity, PPV, and NPV of 0.95–0.97, 0.6, 0.58–0.61, and 0.97–0.98, respectively. The difference in the ability to predict biopsy outcome among studies may be due to differences in the MRI machines and protocols used, as well as to variations in the PIRADS scoring, biopsy protocols, and patient characteristics.

Two recent randomized controlled trials (RCTs) compared the outcomes between prebiopsy MRI with the addition of a targeted biopsy and a conventional TRUS-guided random biopsy [15, 16]. One used a MRI-TRUS fusion targeted biopsy, while the other used the cognitive registration technique, similar to our study. Both found that the PCa detection rate was similar between the MRI and control groups, which is not consistent with our results. Selection bias in our retrospective study could have been the major reason for the difference versus the two RCTs, and patient selection after MRI may have been one of the major causes of the higher cancer detection rate in our MRI cohort. There were also differences in the study protocols, sample sizes, and biopsy protocols among studies. Furthermore, the PCa detection rate of 54–57% for the conventional TRUS-guided biopsy of the two RCTs was higher than that in our study, and another reported study [5].

This study had some limitations. First, the analysis was retrospective and patient selection bias may have been present, as described above. However, it appears based on the statistics that the two cohorts were very similar other than prostate size. Second, PIRADS scoring was not performed at the same time as the biopsy. Third, some patients underwent 1.5-Tesla MRI, whereas others underwent 3-Tesla MRI, reflecting technological changes. However, the PCa detection rate did not differ significantly between 1.5-Tesla and 3-Tesla MRI (data not shown). Fourth, there is no widely accepted definition of clinically significant PCa. Fifth, the promise of targeted biopsies is to reduce the number of total biopsies. However, our study suggests that systematic biopsies should not be omitted when cognitive fusion transperineal biopsies are performed because one third of the tumors were missed by targeted biopsies but detected by systematic biopsies (Fig. 3). Sixth, several radiologists were involved in reporting the MRIs and several urologists performed biopsies. However, this could be deemed an advantage, since clinical effectiveness of prebiopsy mpMRI was demonstrated despite this heterogeneity. Finally, only one radiologist was involved in the MRI review of the PIRADS-2 scoring in each institution; therefore, inter-observer reliability could not be assessed.

## Conclusions

Prebiopsy mpMRI with subsequent systematic plus targeted biopsies could yield a more clinically significant PCa detection rate than a non-targeted TRUS-guided biopsy in biopsy-naïve patients. PIRADS-2 scoring is useful for predicting biopsy outcome. However, large prospective studies are needed to confirm our results.

## Abbreviations

AUC: Area under the curve
CI: Confidence intervals
GS: Gleason score
IQR: Interquartile range
mpMRI: multiparametric magnetic resonance imaging
MRI: Magnetic resonance imaging
NPV: Negative predictive values
PCa: Prostate cancer
PIRADS: Prostate imaging reporting and data system
PIRADS-2: Prostate imaging reporting and data system version 2.0
PPV: Positive predictive values
ROC: Receiver operating characteristic
TRUS: Transrectal ultrasound
